# Serum zinc levels in cancer patients are low and difficult to elevate when complicated by liver cirrhosis: A retrospective study

**DOI:** 10.1097/MD.0000000000032703

**Published:** 2023-01-20

**Authors:** Rie Sugimoto, Yuki Tanaka, Takeshi Senju, Yusuke Morita, Lingaku Lee, Masayuki Hijioka, Terumasa Hisano, Masayuki Furukawa

**Affiliations:** a Department of Hepato-Biliary-Pancreatology, National Hospital Organization Kyushu Cancer Center, Fukuoka, Japan.

**Keywords:** cancer patients, elevated zinc levels, liver cirrhosis, serum zinc levels

## Abstract

In this study, we analyzed blood zinc concentration in patients with various cancer types and the degree of improvement in relation to the underlying disease following treatment with zinc preparations. Serum zinc levels of 530 cancer patients whose blood zinc levels were measured at our hospital from 2016 to 2021 were retrospectively examined in accordance with the primary disease. Changes in zinc levels were analyzed in 155 patients whose zinc levels had been measured on 2 or more occasions in accordance with whether they had received zinc preparations. In addition, the concentration course of zinc before and after zinc formulation administration in 73 patients was examined in accordance with the presence or absence of liver cirrhosis complications. Mean serum zinc levels were below normal in all carcinomas measured, and zinc levels were significantly lower in cirrhosis–hepatocarcinoma cases than in other primary disease cases. Furthermore, serum zinc levels in patients who did not receive zinc preparations decreased significantly over time. In patients who received zinc preparations, the elevated levels of zinc after treatment were significantly lower in patients with cirrhosis than in those without cirrhosis. There was a weak inverse correlation between pre-dose zinc concentration and increased zinc concentration in patients with cirrhosis. In the analysis of covariance, the presence of liver cirrhosis was predominantly correlated with elevated zinc per dose. In summary, serum zinc levels in cancer patients are low and especially low in cancer patients with liver cirrhosis compared with those without cirrhosis after the administration of zinc preparations.

## 1. Introduction

Zinc is classified as an essential trace element, and its bioaccumulation is second only to iron among the 9 essential trace elements in humans.^[1]^ The amount of zinc in the adult human body is 1.5 to 3 g, approximately 60% of which is found in muscle. Organs with high zinc concentrations are the liver, kidneys, pancreas, prostate, and eyes.^[2]^ Zinc is required for the activity of many enzymes, including deoxyribonucleic acid polymerase, ribonucleic acid polymerase, and superoxide dismutase. Zinc plays key roles in many biological functions such as taste, growth, skin metabolism, mental function, and immune function.^[3]^ Zinc has also received attention in relation to cancer. Previous studies reported that zinc deficiency reduces granulocyte migration and phagocytosis and decreases cytokines,^[4]^ which may reduce cancer immunity. Several studies have reported on the blood levels of zinc in cancer patients, with low levels reported in gastric,^[5]^ colorectal,^[6]^ and lung cancer.^[7]^ However, there are few reports comparing zinc blood levels across cancer types.

Zinc absorption mainly occurs in the distal duodenum and proximal jejunum.^[8]^ Absorbed zinc is transported to the liver, where it is taken up and then distributed throughout the body.^[9]^ The relationship between liver function and blood zinc levels has been well studied. Reports have shown that liver fibrosis correlates with low blood zinc levels^[10]^ and zinc administration improves liver reserve capacity.^[11]^ However, whether the degree of increase in blood zinc levels at different doses differs depending on the presence or absence of liver disease is not known.

In this study, we extracted data of blood zinc levels measured in patients with various cancer types and retrospectively analyzed the changes in blood levels in zinc-treated and non-treated patient groups. We also examined changes in blood levels in the treated group in the presence or absence of liver fibrosis.

## 2. Methods

### 2.1. Patients

All cancer patients whose serum zinc levels were measured after 2016 at our hospital were included in the study. A total of 530 cancer patients had serum zinc levels measured. To prevent selection bias, this observational study enrolled all patients who did not refuse to participate in the study. The study was posted on the hospital’s website and posters, and the opportunity to refuse was provided using opt-out, but no patients refused to participate. The patient group included 142 patients with liver cancer, 120 with pancreatic cancer, 68 with gastrointestinal cancer, 33 with biliary tract cancer, 44 with hematopoietic malignancies, 39 with lung cancer, 35 with head and neck cancer, 15 with breast cancer, 11 with skin, bone, and soft tissue tumors, 9 with gynecological cancer, and 6 with genitourinary cancer.

The study was conducted in compliance with the Declaration of Helsinki and with the approval of the ethics committee of Kyushu Cancer Centre (2020-71).

### 2.2. Measurement of serum zinc

Serum zinc levels were measured for all patients using ESPA ZnII reagent (NIPRO Corp., Osaka, Japan).

### 2.3. Data analysis

Changes in zinc levels between 2 time points were analyzed in accordance with the primary disease, the presence or absence of cirrhosis and, for patients whose zinc levels were measured at least twice, whether or not patients received a zinc preparation, the percentage change per elapsed time for patients who did not receive a zinc preparation, and the change per dose for patients who did.

### 2.4. Statistical analysis

Data are expressed as the mean and standard deviation. Statistical analysis was performed using Welch’s *t* test, Student’s *t* test, Fisher’s exact test, Mann–Whitney *U* test, covariate analysis, Shapiro–Wilk test, Cox proportional hazards analysis, logistic analysis, log-rank test, and c-index. All statistical analyses were performed using JMP Pro version 15.1.0 (SAS Institute Inc., Cary, NC), and graphs were generated using PRISM version 9.1.0 (GraphPad Software, La Jolla, CA).

## 3. Results

**Figure 1.**
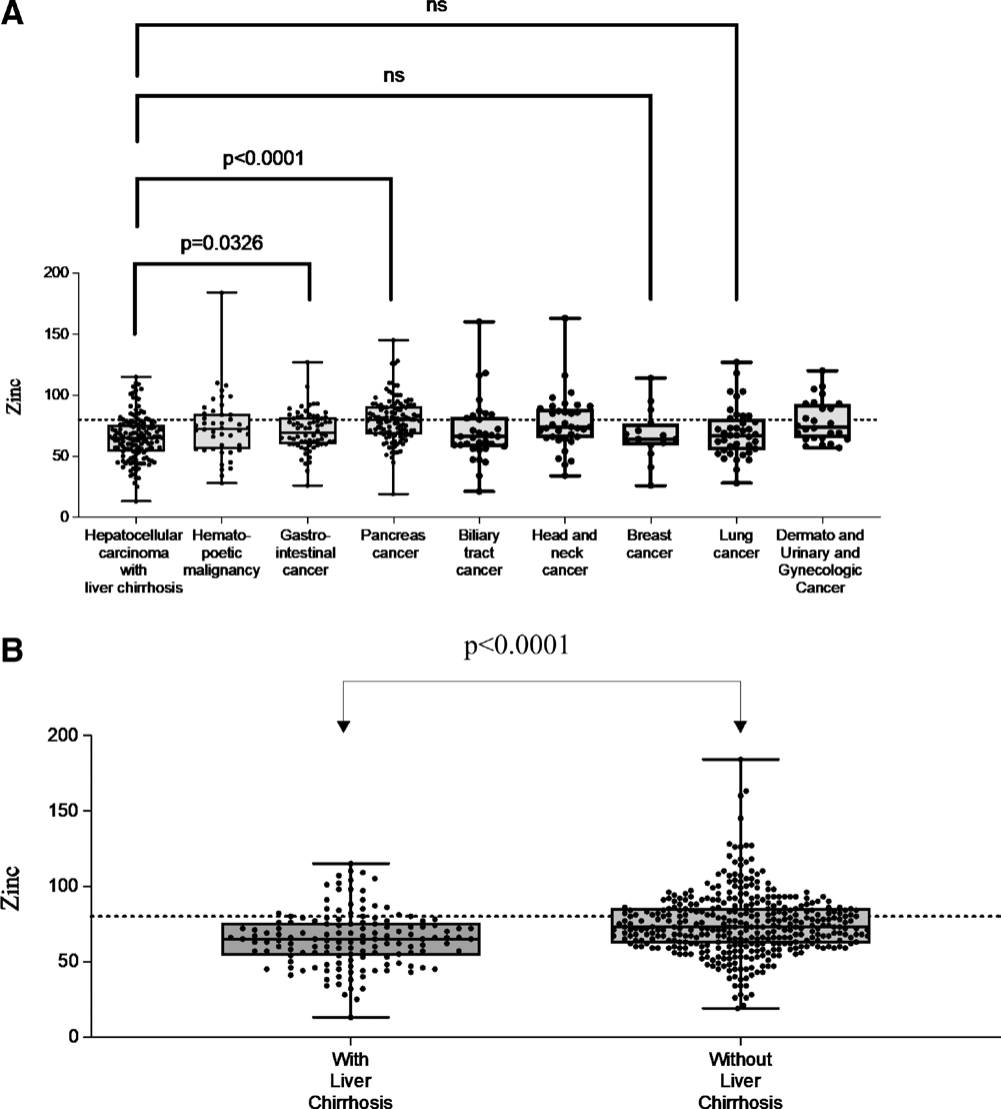
Serum zinc levels by primary disease. (A) Serum zinc levels by cancer type. (B) Serum zinc levels by presence or absence of liver cirrhosis complications. One dot indicates one case, and the dotted line is 80 µg/dL; there is significant difference between arrows.

**Figure 2.**
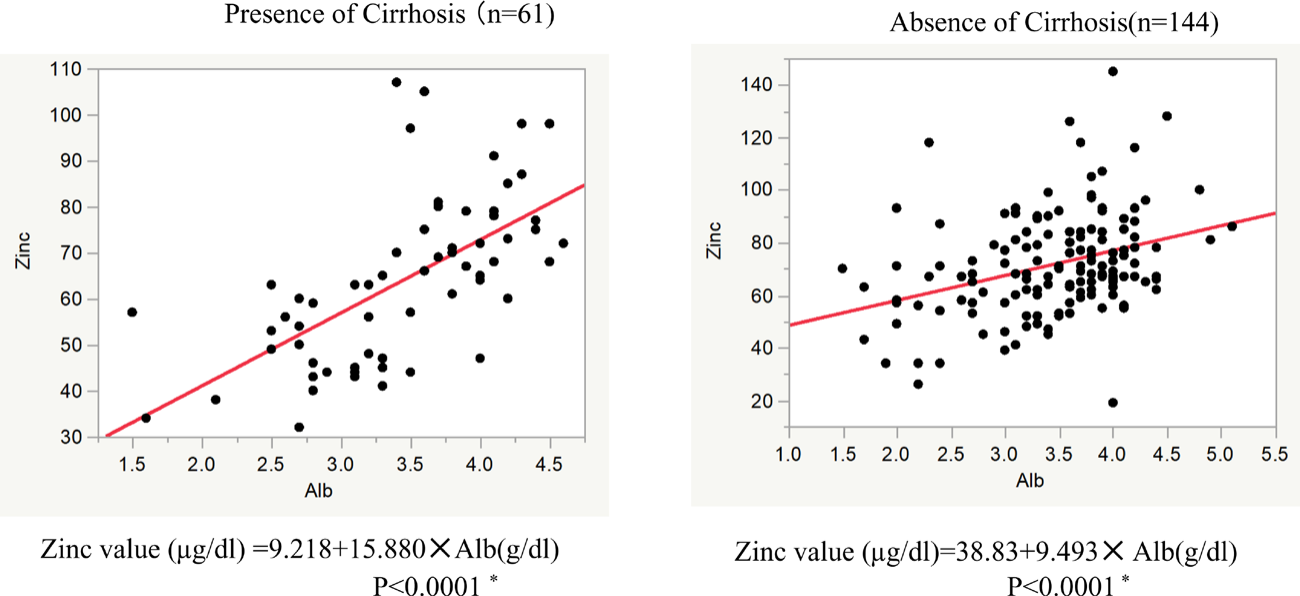
Relationship between serum albumin and serum zinc levels. Left, with cirrhosis. Right, without cirrhosis. Predicted values by single regression analysis; leverage ratio plot, 95% CI. CI = confidence interval.

Zinc levels were measured in 530 patients. Zinc values by primary disease are shown in Figure 1A. The Shapiro–Wilk test was performed for each carcinoma, and the results showed a normal distribution for liver, pancreatic, gastrointestinal, breast, and lung cancers. Head and neck cancer, biliary tract cancer, hematologic cancer, and dermato-urinary cancer did not show a normal distribution; this may be because of the small number of cases of these cancers. The mean serum zinc levels of cancer patients were below normal for all cancer types. The comparison by primary disease was limited to cancer types with normal distribution; the serum zinc levels were lower in cirrhotic liver cancer cases than in pancreatic and gastrointestinal cancer cases, with a significant difference. Serum zinc levels were significantly lower in patients with cirrhosis (*P* < .0001), with 74.7 ± 20.9 µg/dL in patients with cirrhosis (n = 386) and 65.1 ± 18.4 µg/dL in patients without cirrhosis (n = 144) (Fig. 1B). A zinc level below 65 µg/dL, which is considered to suppress T lymphocytes involved in cancer immunity,^[12]^ was observed in 51.3% of patients with cirrhosis and 24.4% of patients without cirrhosis. Albumin levels and serum zinc concentrations were correlated regardless of the presence or absence of cirrhosis, but the strength of the correlation differed between the presence and absence of cirrhosis (Fig. 2).

**Figure 3.**
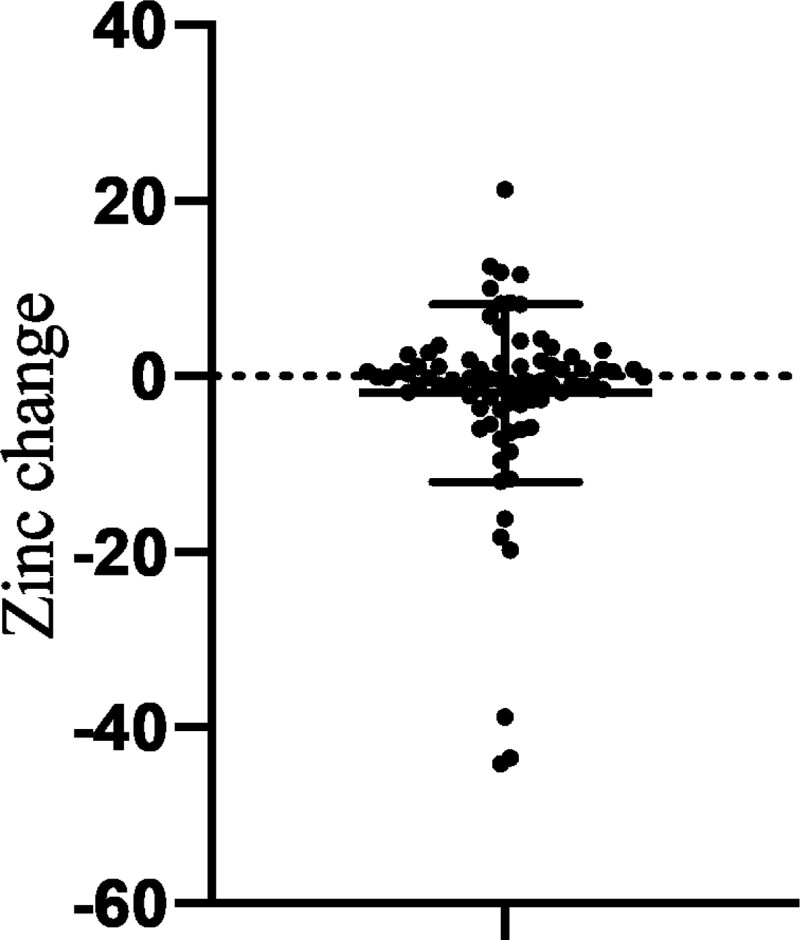
Changes in zinc levels per month without zinc preparation treatment. Zinc levels were decreased with significant difference.

**Figure 4.**
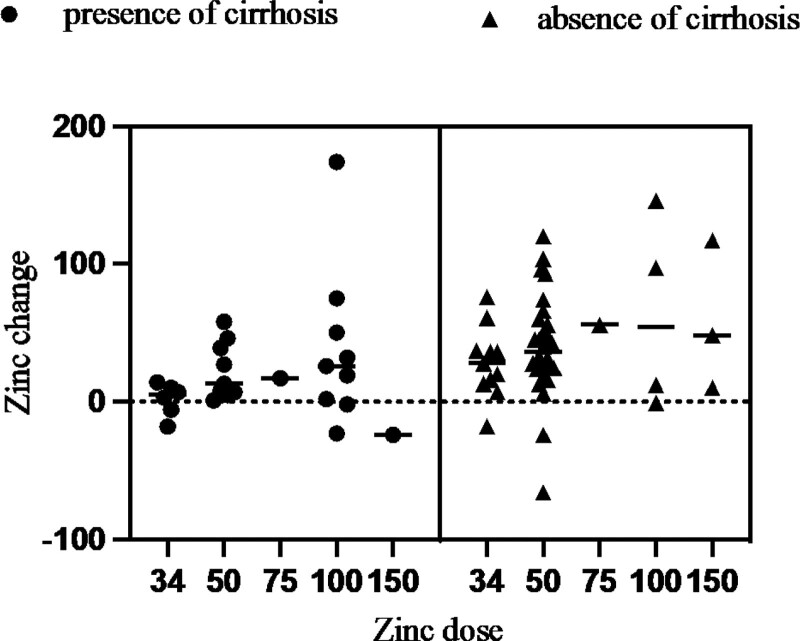
Changes in serum zinc levels after 1 month for each daily dose of zinc. Right lane, without cirrhosis. Left lane, with cirrhosis.

**Figure 5.**
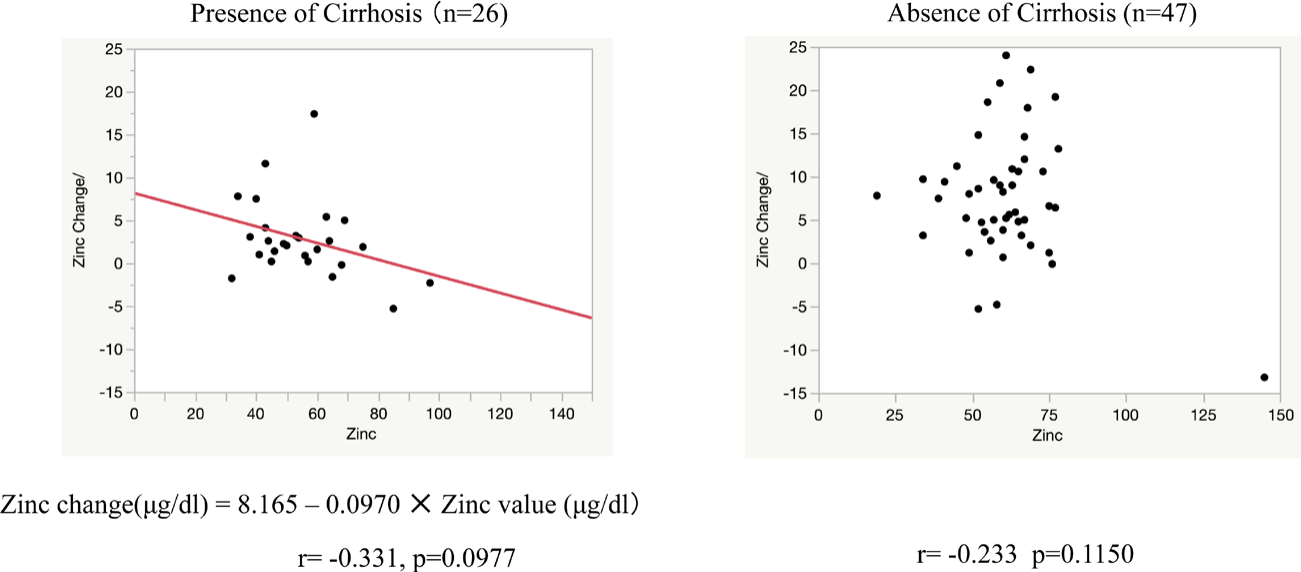
Relationship between serum zinc levels and zinc changes with and without cirrhosis. Scatterplot of changes in serum zinc levels per 10 mg dose of zinc preparation and pre-dose zinc levels and predicted values from correlation and single regression analyses. Right lane, without cirrhosis. Left lane, with cirrhosis.

**Figure 6.**
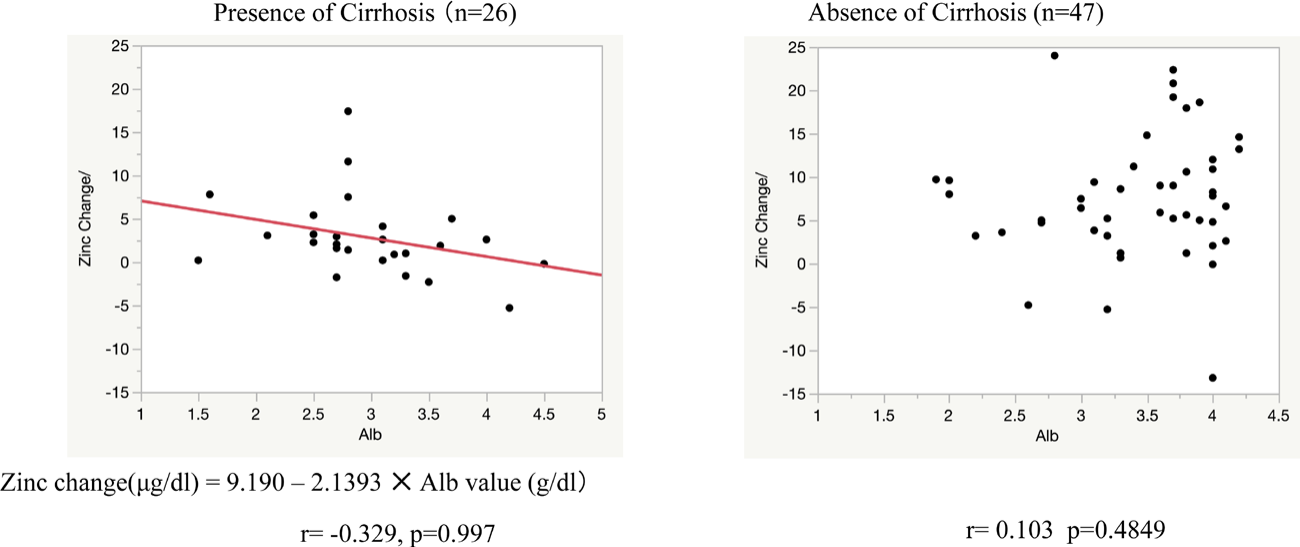
Relationship between serum albumin levels and zinc changes with and without cirrhosis. Scatterplot of changes in serum zinc levels and pre-dose albumin levels per 10 mg dose zinc preparation and predicted values from correlation and single regression analyses. Right lane, without cirrhosis. Left lane, with cirrhosis.

**Figure 7.**
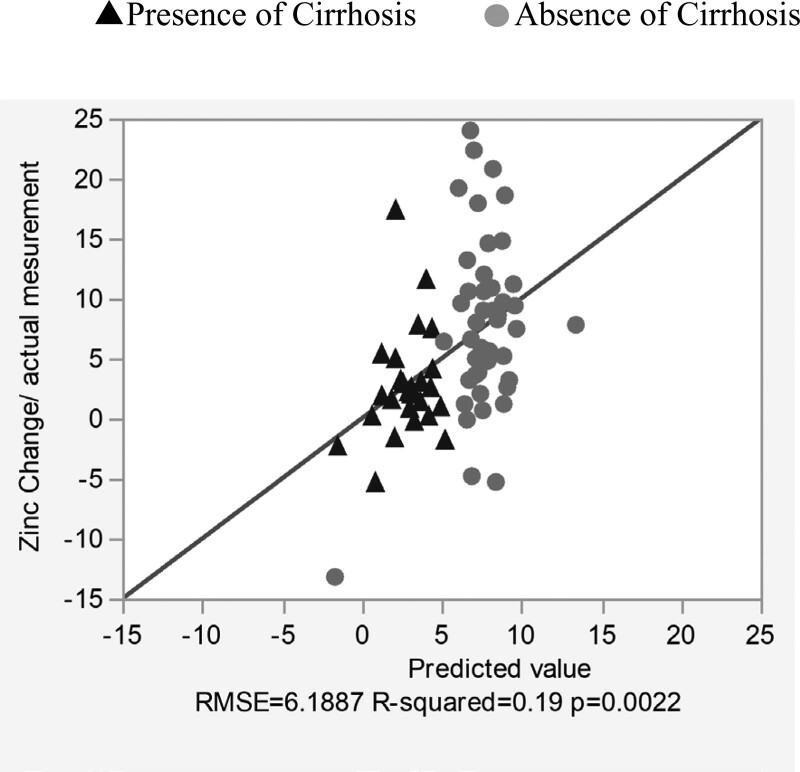
Multiple regression analysis of changes in zinc values. Scatterplot of predicted and measured changes in serum zinc levels per 10 mg dose of zinc preparation. ▲ with cirrhosis, ○ without cirrhosis.

There were 155 cases in which zinc levels were measured on 2 or more occasions: 82 had no zinc product administered during the course of the study and 73 had zinc product administered and levels were measured before and after administration. In patients who did not receive zinc preparations, the blood concentration of zinc tended to decrease over time, with a decrease of 4.34 ± 2.67 µg/dL per month (*P* < .0073) (Fig. 3). Blood levels of zinc were significantly higher in patients treated with zinc preparations than before (*P* < .0001), at + 33.9 µg/dL (95% confidence interval: 4.69–43.2). However, the extent of the increase varied in accordance with the presence or absence of cirrhosis. The increase in serum zinc levels in the 26 patients with cirrhosis was 21.5 ± 7.69 µg/dL, significantly less than in the 43 patients without cirrhosis, 40.0 ± 5.72 µg/dL. Comparison of elevated serum zinc levels at different zinc doses showed that at all doses, the group with liver cirrhosis had a less severe elevation than the group without liver cirrhosis. The median increase in zinc levels in patients with 100 mg/day zinc preparation was significantly lower in patients with cirrhosis (at 26 µg/dL) compared with patients without cirrhosis (54.5 µg/dL) (*P* = .0008; Fig. 4). The degree of increase in zinc per dose showed no correlation in the no-cirrhosis group, but a weak inverse correlation with the pre-dose zinc level in the cirrhosis group was evident (Fig. 5). The relationship between pre-dose albumin levels and elevated zinc showed a weak inverse relationship between albumin levels and zinc per 10 mg dose in the group with cirrhosis and no significant relationship in the group without cirrhosis (Fig. 6). Analysis of zinc and albumin levels before zinc preparation treatment as covariates showed that the presence of liver disease contributed to an increase in zinc per 100 mg/day zinc preparation, with a significant difference (*P* = .0029). The contribution ratio was 97.86%, the logistic regression equation was 7.95 + 2.46 × (no liver cirrhosis) + 1.32 × (serum zinc level µg/dL) − 0.11 × (Alb level g/dL), and the root mean squared error was 6.18 (Fig. 7).

The relationship with blood levels of copper was also examined. Copper levels were measured in 86 of the patients in the no-cirrhosis group and 93 of those with cirrhosis. Blood levels of copper in the group without cirrhosis showed a normal distribution, while levels in the cirrhosis group were not normally distributed. Blood levels of copper were 119 ± 3.98 µg/dL in patients without cirrhosis and 121 ± 3.83 µg/dL in patients with cirrhosis; both were within normal limits. No significant differences in blood copper levels were found between patients with and without cirrhosis (*P* = .700, data not shown). The copper/zinc ratio was further examined. The Cu/Zn ratio in the group without cirrhosis was 1.801 ± 0.095 and that in the group with cirrhosis was 1.994 ± 0.092; both were higher than the Cu/Zn ratio in normal subjects shown in a previous report.^[13]^ Analysis of the Cu/Zn ratio in cirrhotic and non-cirrhotic groups showed that the ratio tended to be slightly higher in the cirrhotic group, but the difference was not significant (*P* = .1469, data not shown). No significant correlation was found between blood levels of copper and the increase in zinc when zinc preparations were administered or between the Cu/Zn ratio and the increase in zinc (*P* = .1383, *P* = .1325, data not shown).

## 4. Discussion

In this study, serum zinc level was measured in a number of carcinomas, and many were deficient in zinc. Zinc levels were particularly low in cancers complicated by cirrhosis. The large overlap between serum concentrations in cirrhotic and non-cirrhotic patients may appear to reduce the clinical significance. However, 51.3% of patients in the cirrhosis group had zinc levels less than 65 µg/dL, which is considered to suppress the activity of T cells,^[14]^ the main immunocompetent cells against cancer. Only 24.4% of patients in the non-cirrhosis group had less than 65 µg/dL, which may represent a significant clinical difference in terms of immune status against cancer. Zinc deficiency is common in patients with liver disease and is correlated with the severity and degree of fibrosis.^[14,15]^ Serum zinc levels decrease in liver disease for the following reasons: (1) a decrease in albumin, transferrin, and other substances from decreased synthesis in the liver, resulting in a decrease in protein-bound zinc and an increase in amino acid–bound zinc, which increases the amount of zinc excreted in the urine^[16]^; (2) portal hypertension associated with cirrhosis causes abnormal hepatic–intestinal circulation, atrophy of the small intestinal mucosa, and zinc malabsorption^[17,18]^; (3) increased urinary excretion of zinc associated with portal vein major circulation shunts^[19]^; and (4) diuretics inhibit the reabsorption of zinc from the tubules.^[9]^ Furthermore, previous studies reported that serum zinc levels decrease with fibrosis even in chronic liver disease that does not lead to cirrhosis,^[15]^ suggesting that wasting because of persistent chronic inflammation may be responsible for the decrease in zinc levels. Metallothionein is involved in zinc homeostasis in vivo,^[20]^ but metallothionein has been shown to be 25% to 30% lower in liver with cirrhosis than in normal liver tissue.^[21]^ Zrt- and Irt-like protein-4 is involved in zinc homeostasis.^[22]^ The present analysis revealed a weak inverse correlation between serum zinc levels before and per-dose increase after zinc administration in patients with cirrhosis, indicating that this maintenance mechanism is preserved even in the presence of cirrhosis. However, the increase in zinc levels after administration of zinc preparations was significantly lower in patients with cirrhosis than in those without. The poor increase in zinc levels with zinc preparation treatment in patients with cirrhosis may be due to impaired absorption of zinc from the small intestine from small intestinal mucosal atrophy and a reduction in metallothionein and other substances required for zinc absorption. Furthermore, because zinc storage takes place in muscle, bone, and liver, it is possible that local stores are also depleted in cirrhosis and that administration is unlikely to lead to increased blood levels. Increased urinary excretion of zinc because of hypoalbuminemia has been suggested as a cause of low zinc levels in cirrhosis,^[16]^ but it did not prevent the administration of zinc preparations from improving blood zinc levels; rather, the opposite was true. In cases of cirrhosis resulting in low albumin, the amount of zinc stored in the liver^[23]^ may be reduced, possibly resulting in increased blood zinc levels. Zinc and copper antagonize each other during absorption from the intestinal tract. The Cu/Zn ratio is higher in cirrhotic patients than in normal individuals and it increases with the progression of cirrhosis. Blood copper levels are high in patients with cirrhosis^[24]^; furthermore, the Cu/Zn ratio is higher in cirrhotic patients than in normal subjects and it increases with the progression of cirrhosis.^[24]^ In contrast, elevated Cu/Zn ratios have been reported in bladder^[25]^ and lung cancers.^[26]^ In this study, Cu/Zn ratios were compared in a number of carcinomas, and Cu/Zn ratios were high regardless of the presence or absence of cirrhosis. However, there was no significant relationship between the degree of increase in blood zinc with zinc preparations and Cu or Cu/Zn ratio, suggesting that the degree of increase in zinc may be because of a different mechanism than absorption, which is antagonistic to Cu.

This study has several limitations. First, it was a retrospective study and not a prospective comparative study. While data of all patients were measured, they are subject to measurement bias because zinc levels were not measured in all hospitalized patients. This poses a threat with regard to external validity. However, analysis of all measured data may capture changes in zinc values that are not restricted to specific situations. Prospective studies should be conducted to verify changes in zinc levels in all cases. Additionally, while the current study focused on blood zinc levels, whether blood zinc levels accurately reflect the amount of zinc stored in the body is unclear. Nevertheless, studies focusing on changes in blood zinc concentration in the presence or absence of cirrhosis are unprecedented and notable.

## 5. Conclusions

Serum zinc levels in cancer patients were deficient in a wide range of carcinomas. Deficiency was corrected by the administration of zinc preparations, but serum zinc levels tended to be less elevated in cases with liver cirrhosis than in uncomplicated cases. Sufficient doses of zinc preparations need to be administered to achieve a clinical effect of zinc administration, especially in cases of cirrhosis.

## Acknowledgments

We thank H. Nikki March, PhD, from Edanz (https://jp.edanz.com/ac) for editing a draft of this manuscript.

## Author contributions

**Conceptualization:** Rie Sugimoto, Lingaku Lee, Masayuki Hijioka, Terumasa Hisano, Masayuki Furukawa.

**Data curation:** Rie Sugimoto, Yuki Tanaka, Takeshi Senju, Yusuke Morita.

**Formal analysis:** Rie Sugimoto.

**Investigation:** Rie Sugimoto.

**Methodology:** Rie Sugimoto.

**Project administration:** Rie Sugimoto.

**Resources:** Rie Sugimoto.

**Validation:** Rie Sugimoto.

**Visualization:** Rie Sugimoto.

**Writing – original draft:** Rie Sugimoto.

**Writing – review & editing:** Yuki Tanaka, Takeshi Senju, Yusuke Morita, Lingaku Lee, Masayuki Hijioka, Terumasa Hisano, Masayuki Furukawa.
